# Nutritional care of Danish medical inpatients: Effect on dietary intake and the occupational groups' perspectives of intervention

**DOI:** 10.1186/1475-2891-3-12

**Published:** 2004-09-13

**Authors:** Karin O Lassen, Filip Kruse, Merete Bjerrum, Lillian Jensen, Kjeld Hermansen

**Affiliations:** 1 Department of Endocrinology and Metabolism, Aarhus Sygehus, Aarhus University Hospital and University of Aarhus, Aarhus, Denmark; 2 State and University Library, University of Aarhus, Aarhus, Denmark; 3 Department of Nursing Science, Faculty of Health Sciences, University of Aarhus, Denmark

## Abstract

**Background:**

Many patients do not eat and drink sufficiently during hospitalisation. The clinical consequences of this under nutrition include lassitude, an increased risk of complications and prolonged convalescence. The aim of the study was 1) to introduce intervention targeting nutritional care for medical inpatients, 2) to investigate the effect of this intervention, and 3) to investigate the occupational groups' attitudes towards nutritional intervention and nutritional care in general.

**Methods:**

The design was to determinate the extent to which the protein and energy requirements of medical inpatients were met before and after intervention. Dietary protein and energy intakes were assessed by 72-hour weighed food records. A total number of 108 medical patients at four bed sections and occupational groups in the two intervention bed sections, Aarhus University Hospital, Denmark participated. The intervention included introduction and implementation of nursing procedures targeting nutritional care during a five-month investigation period using standard food produced at the hospital. The effect of intervention for independent groups of patients were tested by one-way analysis of variance. After the intervention occupational groups were interviewed in focus groups.

**Results:**

Before the intervention hospital food on average met 72% of the patients' protein requirement and 85% of their energy requirement. After intervention hospital food satisfied 85% of the protein and 103% of the energy requirements of 14 patients in one intervention section and 56% of the protein and 76% of the energy requirement of 17 patients in the other intervention section. Hospital food satisfied 61% of the protein and 75% of the energy requirement in a total of 29 controls. From the occupational groups' point of view lack of time, lack of access to food, and lack of knowledge of nutritional care for patients were identified as barriers to better integration of nutritional care into the overall care provision.

**Conclusion:**

There was ample room for improving the extent to which standard hospital food satisfies patients' protein and energy requirements, but implementation of procedures addressing nutritional care were difficult, especially at bed sections with a large staff turnover.

## Background

Many patients do not eat and drink sufficiently during hospitalisation. Thus, 30–50% of the elderly patients are undernourished [1,2] and most of these patients' protein and energy requirements are not met [3,4]. Their muscular tissue, including their heart and respiratory muscles, is adversely affected by this situation [5] and their immune function is suppressed [1,6]. The clinical consequences include lassitude, difficulty in mobilising, prolonged convalescence [1,7] and an increased risk of pressure wounds [8], phlebitis and infections [9,10]. Patients often have reduced appetite, nausea or aversion towards certain types of food, which may partly explain the inadequacy of their food and liquid intake. Intervention studies have shown that by offering food or in-between meals rich in energy and protein, it is possible to increase the patient's protein and energy intake [11-14]. However, most of these intervention studies only use quantitative data. The present intervention study offers data, both quantitative on patients' food intake and qualitative on the occupational groups' attitudes and experiences in relation to the intervention, the food service and the nutritional care in general. These data can contribute to raise our knowledge of nutritional care in general and to identify issues crucial to an improvement of hospitalised patients' food intake in particular.

The first aim of this research was to examine to which extent standard hospital food met hospitalised medical patients' protein and energy requirements. The second aim was to introduce nursing procedures focusing on the nutritional care based on the Danish nutritional recommendations for inpatients [15] to investigate the effect of this intervention on the patients' intake of protein and energy. The third aim was to explore the involved occupational groups' attitudes towards nutritional intervention and nutritional care in general. Particular attention was paid to the identification of problems possibly related to insufficient patient nutrition.

## Methods

### Setting

The setting was an endocrinology ward with 49 beds and 3481 patients discharged during 2002 (divided into bed sections IA and IB) and a cardiology ward with 53 beds and 4542 patients discharged during 2002 (divided into bed sections IIA and IIB) [16]. All hospital food was produced in a central hospital kitchen and transported in heated containers to the bed sections where it was portioned out and served to the patients.

### Design of the study

Medical patients' pre-intervention dietary protein and energy intakes were assessed by 72-hour weighed food records [17] at four bed sections (two wards) to include the appropriate number of patients. Before the intervention the bed sections at each ward was randomised to intervention or control. After a five-month intervention period, patients' dietary protein and energy intakes were assessed to evaluate the effect of intervention. After intervention the occupational groups involved in the nutritional care and the food service at the two intervention sections were interviewed in focus groups or by individual interview.

### Participants

Both acute and referred medical patients at all ages participated. The inclusion criteria were defined as: 1) the patient was not placed on a prescribed diet, 2) the patient had no contact with, or had not previously received dietary advice from a clinical dietician, 3) the patient did not belong to an ethnic minority, and 4) the patient was hospitalised for at least five days. Patients with dementia and patients who were severely mentally or physically impaired were excluded.

Typical patient diagnoses included acute or chronic lung disease (e.g. chronic obstructive lung disease, asthma, bronchitis), acute or chronic cardiovascular disease (e.g. hypertension, angina, thrombosis, apoplexy), metabolic disorders (e.g. thyrotoxicosis, osteoporosis) or infectious disease (e.g. pneumonia, cystitis). The nursing staff selected the patients meeting all the criteria. The patients received oral and written information about the investigation underlining the voluntary nature of their participation. Three or four patients from a bed section participated at the same time, providing data for the food records.

The occupational groups participating in the focus group interviews were: nurses, health care support staff and nurse aides on day or evening duty from one of the two intervention sections IB and IIB (four interviews), two nurses in charge from the two intervention sections (one interview), three maids from the two intervention sections (one interview), two clinical dieticians from the two intervention sections (one interview), and one catering officer from the kitchen (one individual interview). In total 26 informants participated in eight interviews.

### Patients' characteristics

Data on patient age, date of and diagnosis on hospitalisation, second diagnosis, oedema, dehydration, body weight on hospitalisation (if measured) were collected by the investigators from hospital records. Body temperature (if fever) was collected from the hospitals records during the 72-hour food recording. Patients' body weights were recorded twice: at hospitalisation (or when they were included in the study) and on discharge. This weighing was standardised according to time of the day, the patients dress and the scales. The changes in body weight during hospitalisation were recorded for the patients not having oedema or dehydration. Patient height was measured and body mass index (BMI, kg/m^2^) calculated. The patient was asked about ability to chew and swallow and recorded as 'effortless', 'slight difficulty' or 'with difficulty'. On discharge they ascribed to the meals during hospitalisation was recorded as 'very important', 'of some importance', 'almost no importance' and 'no importance'.

### Food records

The patients had their food and drink weighed for 72 hours at breakfast, lunch, afternoon coffee and supper by the investigators. The patients, relatives or staff recorded the last-meal-of-the-day and individual between-meals as estimated records. The investigators contacted the patients three to five times a day to follow up on these estimated records. The weight of the between meals provided by visitors was estimated by weighing similar food items.

The food items were weighed in the form received from the kitchen. Potatoes, mashed potatoes, sauce, meat, etc. were weighed separately. Standardized menus such as stew, open sandwiches, sandwiches, etc. were weighed in full. The total weight of the food items each patient was served was weighed before serving as was plate waste after the meal. Drinks were estimated and recorded when poured into a glass or feeding cup one centimetre below the rim.

### Dietary intake of protein and energy

24-hour food records were checked and coded to calculate protein (gram) and energy (kJ) intake by the investigators. The calculations were based on the data from the recipes used in the hospital kitchen and the Danish Database 'Dankost 2000', which contains data from the Danish food tables [18].

### Physical activity

Each patient's physical activity was recorded every hour during three days and nights (72 hours) in the period of food recording. It was recorded whether the patient was 'lying asleep', 'lying awake', 'sitting', 'walking' or 'training'. For walking or training the approximate duration of activity was recorded as a fraction of an hour, and a factor of physical activity was estimated for each 24-hour period [15]. The investigators contacted the patients three to five times a day to follow up on the recording of the physical activity.

### Estimation of protein and energy requirements

Official Danish food recommendations for institutions propose that patients with chronic diseases have 1.0–1.5 gram of protein per kilogram body weight depending on the degree of stress metabolism [15]. A factor of 1.2 gram was used as an estimate of moderate metabolic stress [19,20]. However, they did not allow for the underestimation of underweight and overestimation of overweight patients' requirements. The calculations were adjusted accordingly in the following way: If the BMI was below 20, the recommended requirement was calculated as 1.5 gram per kilogram bodyweight per 24 hours. If the BMI was above 30, the recommended requirement was calculated as 1.0 gram per kilogram bodyweight per 24 hours [21].

The estimated energy requirement was calculated as 'basal metabolic rate' (Harris Benedict equation [22]) x 'the factor of physical activity' x 'the factor required to increase the body weight (if the BMI was below 20)' or a 'factor of stress (if the BMI was above or equal to 20)' [15,21]. If the BMI was below 20, the factor 1.3 was used instead of the stress factor. The factor required to increase the body weight was an estimate of the amount of energy the patient was able to consume [15,22]. The stress factor was applied in patients judged to have metabolic stress because of their pathological condition. The stress factor range was 1.1–1.4 for patients with chronic lung disease, chronic heart disease and apoplexy: severe infections were given a factor of 1.3. The stress factor was determined by the temperature and was set at 1.2 at a temperature of 38°C, 1.3 at 39°C and 1.4 at 40°C. Only one factor of stress was used, and the temperature stress factor had the highest priority [15].

The mean recorded protein (g) and energy (kJ) intake was compared with the estimated protein (g) and energy requirements (kJ), and the degree (in percent) to which the patient's 24-hour requirements were met.

### Intervention

The nurses in charge from the two intervention bed sections IB and IIB received information a) specifying to which degree the patients' protein and energy requirements were being met before intervention and b) detailing the Danish Recommendations for Hospitalised Patients [15]. In order to introduce and facilitate continuous staff monitoration of the patients' nutritional status during their hospitalisation the following intervention procedures were formulated in collaboration with the two nurses in charge. Such monitoration would allow the staff to identify patients at risk of under nourishment and would secure continuous registration, which was seen as a precondition for optimising the patients' uptake of nutrients. The procedures were formulated as one standard applying to be used to all non-diet patients admitted to bed sections IB and IIB: *The patient's nutritional status is assessed on admission and during hospitalisation.*

As recommended by the nurses in charge three forms (A, B and C) with different purpose were made to support the staff in relation to the nutritional care.

In **Form A **patient data related to the nutritional care were collected upon admission: height, body weight, BMI, usual body weight and changes in body weight for a defined period (if possible), oedema or dehydration, the date and by whom the patient had been informed about the food service, the result of the first assessment of the nutritional status (result from form B), a short description of 1) the patient's ability to eat and drink, and 2) of the action taken by the staff 3) the date of the next assessment of the nutritional status. The form also allowed room for the results of the next five assessments of the patient's nutritional status.

The purpose of **Form B **was to assess the patient's nutritional status/risk score and suggesting the action the staff could take. Actions were performed according to detailed English Standards [23]. The assessment parameters were body weight for height (BMI), appetite and ability to eat. The patient was assessed at 'low risk' when BMI was normal, the appetite good and the patient fully independent. The patient was assessed at 'moderate risk' when underweight but stable, the appetite poor, and the patient needed help with feeding or had some swallowing difficulties. The patient was assessed at 'high risk' when severely under weight or actively lost weight, ate very little or have had no food for the last four meals, and was dependent on others for feeding or had severe swallowing difficulties. Form B allowed the patient's nutritional status to be recorded six times to ensure continuity of the assessment of nutritional status. A short guide to action was provided to the staff for each of the assessment categories; For the 'low risk' patient 'no action necessary, but check weight weekly'. For the patient at 'moderate risk' the action could be 'check weight weekly, encourage with eating and drinking, replace missed meals with supplements and repeat score after one week and ask medical staff to refer patient to clinical dietician if no improvement'. For the patient at 'high risk' the action was to 'focus on encouraging with eating and drinking, replace missed meals with supplements and repeat score after three to four days and ask medical staff to refer patient to clinical dietician'.

In **Form C **the estimated record of the patient's protein and energy intake could be calculated and compared to data in the nutritional handbook describing what the food items contained of protein and energy. This handbook contained a standardised description of all meals delivered from the kitchen in household measurements (spoons, pieces, decilitre, etc.) and the estimated protein (g) and energy (kJ) content.

### Introducing the standard

The investigators convened meetings with the nursing staff and the domestic helpers at the two intervention sections IB and IIB. The rationale of the standard was explained detailed and both oral and written instructions about the use of the forms were given. Four meetings were held in bed section IB and six in bed section IIB. At these meetings problems, ideas, etc. related to the standard and the forms were discussed and adjusted according to these discussions. The investigators contacted the staff in bed sections IB and IIB once or twice a week during the five-month investigation period to give support if wanted.

The intervention at the two bed sections had no influence on the food production in the hospital. But before the intervention the kitchen produced a 'unrestricted diet' to all patients not placed on a prescribed diet, which contain about 8250 kJ and 70–80 gram of protein with about 15, 41 and 43% of energy from protein, fat and carbohydrates [24]. During the intervention period the kitchen changed the production to two different diets to meet the Danish Nutritional Recommendations for Diseased People [15]; From the kitchen the diets were introduced in the following way; one diet for the elderly and people with little appetite – the so-called 'hospital diet' – and one diet for all patients with ischemic heart disease and diabetes mellitus – the so-called 'normal diet'. The 'hospital diet' contained about 10000 kJ and 90 gram of protein with 18, 40 and 42% of energy from protein, fat and carbohydrates. The 'normal diet' contained about 9000 kJ and 80 gram of protein with 10–15, 30 and 55–60% of energy from protein, fat and carbohydrates [24]. The changes in the diets were introduced to the staff by the clinical dieticians. Besides these diets different commercial and no-commercial protein- and energy supplements, stewed fruit, soup etc. were available from the kitchen.

### Statistical methods

The number of patients required was calculated in the following way:The clinically relevant difference between the average extent to which the patient's protein and energy requirement was met before and after the intervention was estimated to 15% [25]. Patients' dietary protein and energy intakes were estimated to lie 0–50% below their requirements (standard deviation (SD) 12.5–15.0%). A 5% significance level was chosen and the power was chosen to lie at 90%. The t-test was used to calculate an appropriate sample size for the control and intervention groups, viz. a minimum of 21 patients.

The dietary protein and energy intake was calculated as a 24-hour mean (SD) for each patient and for each group (SD) of patients at each bed section. The outcome measure was the percentage degree to which the patient's actual protein and energy requirement was covered compared with his/her estimated requirement. Confidence intervals for the outcome measures were estimated.

The effect of intervention for independent groups of patients were tested by one-way analysis of variance (ANOVA) using the SPSS version 9.0. The assumptions of independence, normality and identical variances were fulfilled. Analyses of covariance were described for non-comparable variables for the four patient groups after intervention.

### The interview in the occupational groups

An interview guide was designed for each of five occupational groups: 1) nurses, health care support staff and nurse aides (four interviews), 2) charge nurses, 3) maids, 4) clinical dieticians and 5) one catering officer from the kitchen [26]. In the interview the investigator focused on the informants' actions, attitudes, experiences and reflections in relation to the intervention and nutritional care. Focus group interviews were considered the most appropriate form of data collection given the intent of the study [27]. All the 26 informants shared experience from the intervention study and from the situations where patients' meals were served. Eight focus group interviews were carried out at the hospital during the working hours in rooms familiar to the voluntary informants. The focus group interviews were tape-recorded with the permission of the informants, who were informed that they could read the transcribed interview, should they wish so. The qualitative data were analysed as a text.

### Ethical approval

The study fulfilled the declaration of Helsinki II and was approved by the Local Scientific Ethics Committee.

## Results

### Patient characteristics

Food records were completed for 48 patients before and 60 patients after intervention. Table 1 summarises the baseline characteristics of the participating patients. The patient groups were comparable with regard to BMI, stress factor and ability to chew and swallow. The average age of the medical patients was 72 ± 11 years. Before the intervention 17 patients out of 22 lost body weight. After the intervention 20 patients out of 37 lost body weight (table 1). Before the intervention, 56% of the patients participating in the study were weighed on admission (defined as within 48 hours from their arrival to the bed section). After intervention 52% of the patients in the control sections and 45% in the intervention sections were weighed on admission by the staff.

**Table 1:** Summarised baseline characteristics of participating patients before and after intervention. Values are group averages (standard deviation (SD)) unless otherwise stated.

	Medical ward I		Medical ward II	
	
**Before intervention**	Bed section IA *Status*	Bed section IB *Status*	Bed section IIA *Status*	Bed section IIB *Status*
Number of patients (women/men)	12 (5/7)	12 (10/2)	10 (7/3)	14 (12/2)
Age, years (SD)	74 (13)	68 (14)	72 (7)	70 (10)
Length of stay, 24 hours (SD)	25 (21)	33 (29)	24 (18)	25 (18)
BMI, kg/m^2 ^(SD)	26.4 (4.2)	26.6 (5.1)	26.1 (6.2)	24.7 (6.0)
BMI, women, kg/m^2 ^(SD),	27.5 (4.2)	27.0 (5.5)	26.5 (6.8)	25.3 (6.2)
BMI, men, kg/m^2 ^(SD),	25.7 (4.4)	24.4 (1.5)	25.2 (5.7)	21.1 (2.5)
Change of body weight per 24 hours, gram (SD) (n)#	20 (100) (4)	-78 (83) (7)	-154 (107) (5)	-6 (174) (6)
	Medical ward I		Medical ward II	
	
**After intervention**	Bed section IA *Control*	Bed section IB *Intervention*	Bed section IIA *Control*	Bed section IIB *Intervention*

Number of patients (women/men)	16 (9/7)	14 (10/4)	13 (9/4)	17 (7/10)
Age, years (SD)	74 (12)	73 (13)	71 (9)	73 (9)
Length of stay, 24 hours (SD)	26 (20)	24 (17)	14 (7)	16 (10)
BMI, kg/m^2 ^(SD)	22.2 (6.3)	22.1 (3.7)	25.9 (7.1)	24.9 (4.9)
BMI, women, kg/m^2 ^(SD)	21.8 (4.7)	22.1 (4.3)	26.6 (8.4)	21.9 (4.5)
BMI, men, kg/m^2 ^(SD)	22.6 (8.0)	22.3 (2.2)	24.4 (3.6)	27.0 (4.0)
Change of body weight per 24 hours, gram (SD) (n) #	-72 (184) (8)	11 (108) (11)	-105 (140) (8)	-87 (238) (10)
Number of patients receiving 'Hospital diet'/'Normal diet'	10/6	11/3	9/4	10/7

# Patients who take diuretics and patients with dehydration or oedema are excluded.

### Patient requirement and protein and energy intake

In table 2 the average degree to which protein requirements were met before and after intervention are summarized. In table 3 the corresponding figures for energy requirements. There were no significant pre-intervention differences between the groups concerning the average degree to which their estimated protein (p = 0.918) and energy (p = 0.367) requirements were met.

**Table 2:** Dietary intake of protein, estimated requirement of dietary protein and degree to which need for dietary protein per 24 hours was covered before and after intervention. Values are group averages (standard deviation (SD)) unless otherwise stated.

	Medical ward I		Medical ward II	
**Before intervention**	Bed section IA *Status*	Bed section IB *Status*	Bed section IIA *Status*	Bed section IIB *Status*

Dietary intake of protein in grams per 24 hours (SD)	63 (26)	56 (10)	55 (20)	59 (23)
Estimated need for dietary protein in grams per 24 hours (SD)	85 (13)	79 (12)	79 (18)	84 (13)
Estimated need for dietary protein covered in per cent (SD)	**73 **(27)	**73 **(15)	**71 **(24)	**72 **(30)
95 % confidence interval	56–90	63–82	54–89	55–89
	Medical ward I		Medical ward II	
	
**After intervention**	Bed section IA *Control*	Bed section IB *Intervention*	Bed section IIA *Control*	Bed section IIB *Intervention*

Dietary intake of protein in grams per 24 hours (SD)	44 (20)	61 (26)	49 (15)	49 (20)
Estimated need for dietary protein in grams per 24 hours (SD)	74 (12)	72 (13)	81 (17)	85 (14)
Estimated need for dietary protein covered in per cent (SD)	**60 **(26)	**85 **(31)	**62 **(19)	**56 **(19)
95 % confidence interval	46–73	67–102	51–74	46–66

The results of the intervention was different at bed section IB and IIB; The intervention significantly improved the degree to which the energy and protein requirements were met among patients in intervention section IB compared with patients in the control sections IA and IIA (protein p = 0.009 and energy p = 0.010). On average, the former had an intake of 85% of their calculated protein requirement and 103% of their energy requirement. In intervention section IIB, the patients only had an intake reaching 56% of their protein and 76% of their energy requirement. These values were on average much lower than for patients in section IB and they were comparable to those obtained in the control sections IA and IIA. Analysis of co-variance for the non-comparable variables age, patient mobility, BMI, type of diet and number of bed-days showed no significant effect on the outcome measure for the degree of meeting the patients' requirement of protein and energy. The patients ability to chew and swallow, and the importance of the meals to the patients during hospitalisation were comparable in the four groups of patients before and after the intervention.

In the control sections the diet met 61% of the patients' protein and 75% of their energy requirements after intervention. These levels were not significantly different from those recorded before the intervention, but 11% and 14% lower than before the kitchen changed the diets.

### The intervention and the occupational groups

During the intervention period, the nursing staff in bed section IB used the forms for assessing the nutritional care of three patients. In intervention section IIB the forms was used assessing the nutritional care of 17 patients. The patients nutritional status/risk score were not determined otherwise.

Analysis of the qualitative data from the eight interviews extracted five templates with questions relevant to an increased risk of insufficient nutritional care:

1. Divergent attitudes towards intervention.

2. Lack of flexibility during meals.

3. Lack of knowledge about nutritional care for patients.

4. Nutrition – a subordinate part of the care.

5. Lack of recognition of responsibility for nutritional care.

### Divergent attitudes towards intervention

Analysis showed that the staff in the intervention sections had not been using the nutritional records systematically. Several nurses thought that the records were too comprehensive and overwhelming. Many mentioned that they had not had the time to learn how to use the records and they were clearly perceived as an extra workload. The nurses in charge mentioned that it was not unproblematic to burden staff with material they did not have the time or resources to read. However, a few staff members, among them two nurse students from bed section IIB, had learned how to use the records. They found that they were utilizable and easy to use.

The two nurses in charge had divergent views on the usability of the intervention study. The charge nurse in bed section IIB thought that the intervention had improved their work with the patients' nutrition. The staff had previously accepted that patients would lie without eating for seven to ten days. Intervention caused the staff to use a feeding tube on threatened patients earlier than before the intervention. However, the charge nurse from bed section IB declared that the staff in her section had not shown much commitment to the intervention. The staff had not taken 'ownership' of the intervention study because the decision to participate in the project had not been a staff decision but one taken by the central management. She emphasized that the staff's attitude was rooted in the fact they had to take in new ideas and instructions all the time.

Several care providers in bed section IB thought that it was a sizeable extra workload to use the records for recording patients' nutritional statuses and that this had constituted a barrier to their active participation in the process. Other nurses in bed section IB declared that they did not think that it was necessary to continuously register a patient's nutritional status. It sufficed for some nurses to use their 'clinical judgement' and on this basis monitor the patient's weight status. These nurses were not interested in any new initiatives and in tools for nutritional care.

The records were not – and are still not – an integral part of the nutritional care in the intervention sections. This impacted on care continuity. The few staff members who had actually been using the records and had been able to identify patients at risk of insufficient nutrition reported that their observations had not been translated into action.

Although the food records were only used to a minor extent, the staff generally agreed to the relevance of focusing on the patients' nutrition. Several nurses had not previously paid much attention to the patient's nutrition, but the intervention had made them more conscious of this issue:

"I must say that after we have begun to pay attention to the diet, it has become clear to me how important it is. You have always known that it was important, but you do not really expect the patients to be undernourished when they are hospitalised" Nurse

After the intervention the nurses were more conscious of their choice of food rich in energy than "*before where they did not pay much attention to the fact that febrile patients constituted a special group at risk of falling into nutritional deficit". *The general belief that 'fat is bad' for patients was widespread before the intervention. This belief springs from general dietary recommendations for healthy people. However, the intervention raised consciousness of the fact that public dietary recommendations may be suitable for healthy, but not for ill people.

### Lack of flexibility during meals

The focus group interviews overall showed that the concept of 'individual nutrition' was not easily introduced in the nutritional practice at the two bed section. The staff was able to offer food five times during 12 hours during a 24-hour period. The duration of the meals was dictated by tight time schedules for maids and the hospital orderly. Lunch and dinner were often served under time pressure. Between the fixed meals, the care providers often lacked the time to offer patients various kinds of between-meals in the form of frozen, heated food. Assuming that nutritional care rests on the efforts of a committed staff, it may be claimed that the very organisation of the food service was counterproductive to individual nutritional care because the staff did not have real opportunities to offer the patients any food outside the fixed meal times. Individual nutritional care was also hampered by the fact that the kitchen ran a 24-hour nutrition schedule. This makes it difficult for the patients themselves to decide which meals they want to eat and hence to involve them in their own nutritional care. This was especially a problem for elderly nibblers.

### Lack of knowledge about nutritional care for the patients

The clinical dieticians disseminated knowledge about nutrition to the staff in the bed sections, e.g. knowledge about a change in diet from 'unrestricted diet' to 'normal diet and 'hospital diet'. However, such knowledge dissemination was obstructed in several ways. A large staff turnover in some sections meant that such knowledge did not stay in the sections. The exchange of permanent nursing staff during a seven month period including the intervention was 9% and 46% in intervention section IB and IIB respectively (Information from the administration, Aarhus University Hospital). It was difficult for the clinical dietician to get through to the entire staff, as those who were willing to listen were those who took interest in the patients' diet:

"But those we do see are those among the staff who take active interest. It's the old guard turning up" Clinical dietician

Some care providers found that it was time-consuming to acquire knowledge about nutrition. Thus a member of the health care support staff mentioned that *"just learning what a 'normal diet' and a 'hospital diet' is takes so much time".*

Along this line, several staff members, especially nursing assistants and health care support staff mentioned that it would be very useful if they had a resource agent they could ask about nutritional issues. It was possible for the care providers to refer the patients to the clinical dieticians. However, they felt that the dieticians were often so busy giving advice to referred patients that they could hardly assume a role in the daily nutritional care. The clinical dieticians, on their side, indicated that they would like the care providers to involve them more so that they could also give advice to patients who had not been referred. However, it was difficult for the clinical dieticians to be allowed to contribute:

"The nurses think that they can manage the patients' nutritional situation. I think that is what they believe today. But if we were there when a question was raised, then they would use us. That's what I think" Clinical dietician

Much would be gained, according to the clinical dieticians, if the staff knew that the patients' loss of weight during hospitalisation should be avoided and if such knowledge was used in the nutritional care.

One nurse put forward the view that recommendations for healthy people also applied to patients. But during the intervention she expressed that she had changed her perception, but she found it difficult to manage nutritional requirements of ill patients and at the same time relate to dietary advice to healthy people, which were also used in the nutritional care of hospitalised patients. The clinical dieticians had also noted that many elderly patients were served the 'normal diet' even if they needed 'hospital diet'. These observations could signal the existence of a gap between the knowledge the nurses had and the knowledge actually needed to asses, among others, which diet suited particular patients best. The introduction of two diets caused some confusion and uncertainty among the occupational groups involved.

### Nutrition – a subordinate part of the care

The care providers expressed an interest in the patients' nutrition, but also mentioned that they often had to ignore this aspect of care because of their tight work schedule. Some days when they had the time and the resources, they would pay more attention to the patients' nutrition, overseeing for example how much the patients were eating. But on busy days the care providers had to abstain – e.g. from offering the patients an extra portion "*because it's nutrition and similar things which we must choose not to include when we are busy".*

Time was a limiting factor in nutritional care. The overall message was that the staff found it difficult to find time for determining the patient's height, calculate BMI, talk with the patient about losing weight, the patient's wishes for diet and his/her possible problems with eating and drinking. Time was hence both a real and an imagined barrier to recording the patients' nutritional status and to including the patients in their own nutritional care.

In relation to that several care providers in section IB said that the nutritional care was a secondary priority. Hence, it was not perceived as a part of the care and treatment itself, but rather as a service "*along with laundering and ironing"*, as mentioned by the charge nurse in section IB. Other care providers in section IB also said that serving food and beverages for the patients was not part of their job:

"You feel you are in the catering business in some way when you have to wait on the patients" Nursing assistant

This would seem to suggest that for some care providers, nutritional care and the tasks such care demanded was not perceived as a natural part of their care activities. If a part of the staff defined nutritional care and the task of making sure that the patients got enough to eat as a job function outside the normal realm of their occupation this evidently constituted a barrier to an improvement in the patients' nutritional care.

The patients' nutrition was hence not a priority area within the overall care work performed by the nurses and it was not an active part of the treatment. Inversely, the nurse in charge in section IIB thought that nutrition should be a first line priority to ensure that the work performed by the other occupational groups could have optimal effect. She pointed, among others, to the restoration of physical strength among stroke patients. She was aware that the work routines and the barriers to knowledge dissemination to other occupational groups was a factor limiting the speed with which changes could be implemented. The nurses in charge' attitudes to intervention and nutritional care were reflected in the attitudes of the rest of the staff.

The data paradoxically showed that although nutritional care falls within the nurses' competence area, they only engaged in such care when they had the time to do so. When the nurses were busy, which they often were according to themselves, they gave lower priority to nutritional care. However, continuity of nutritional care was particularly important in nibblers according to the clinical dietician:

"It is not always big science or intricate calculations; it's almost just a simple matter of remembering to serve the food to the patient" Clinical dietician

### Lack of recognition of responsibility for nutritional care

The results suggest that the occupational groups involved in the food service had different guidelines. The assistant catering officer from the kitchen declared that she adopted a 24-hour approach to the planning of menus and distribution of energy percentages. The care providers prioritised that patients ate the meals they chose from the menus. However, this target was compromised by constraints of time and choice. The maids stuck meticulously to the diet previously decided by the nurses for each individual patient, while the care providers did not. Moreover, contrary to the clinical dieticians, some care providers thought that overweight patients should lose weight during their admission. The dietary change introduced to make some of the patients lose weight was, however, criticized by some of care providers in section IIB. They found that the change to 'normal diet' was not clear to the patients and was an expression of abuse of power, because the patients did not have any choice.

The data gave no indications that the involved occupational groups shared a common goal as far as nutritional care was concerned. Inversely, the different groups had different priorities and showed neither insight nor any understanding of the professional competences of the other groups. The clinical dieticians also mentioned that a relatively high staff turnover at the bed sections ran counter to continuity of nutritional care and made it difficult to maintain a high, constant level of nutritional knowledge at the bed section derived through instruction and teaching undertaken by the clinical dieticians.

Furthermore, the responsibility for the practical aspects of nutritional care could not be precisely located because many different staff groups were involved. This invariably increased the risk that responsibility was diluted, viz. that the individual care provider loses his/her sense of responsibility and overview of the situation. The staff in the bed sections did not see precise definition of responsibility as a central issue as opposed to staff outside the bed sections who would like to see a clear formal distribution of responsibility for nutritional care with a view to improving communication and procedures.

"Nutritional care, distribution and orders should be given priority from above. It should not be the maids who should work for this. They are often having all the problems because the care providers have other duties they must see to; so the maids are sometimes doing as best they can; and what else can they do? But it should be a priority coming from the very top" Assistant catering officer

The data suggested that dilution of responsibility was accompanied by an element of responsibility evasion. The care providers are, theoretically, responsible for the patients' nutritional care, but the maids assumed the lion's share of this responsibility in practice. The maids were employed in the maintenance section and therefore had no occupational responsibility for the patients' nutrition. However, the maids were very committed and felt responsible for the patients' nutrition. They found it difficult to accept that they had no guarantees that other staff members would take responsibility for the patients' diet when they were not at work. When the maids were having their weekends, holidays etc., the substitutes would often take over their function. The maids declared that they would be happy to take a more active role in the patients' nutritional care. Through their teaching at the bed sections, the clinical dieticians had learned that the maids in general were showing much commitment, attention and responsibility towards the issue of the patients' nutritional status. Inversely, several care providers found it difficult so see themselves take a more active role vis-à-vis nutritional care.

As a measure intended to counteract the dilution and evasion of responsibility, the assistant catering officer suggested that central hospital management should issue a clear statement that the patients' nutritional status was a high priority area that deserved serious attention from all occupational groups. Such a message could also give impetus to a process of clarifying responsibilities and tasks related to nutritional care in all bed sections.

## Discussion

Prior to intervention food ingested during hospitalisation on average met 72% of the patient' protein (table 2) and 85% of their energy requirement (table 3), and there was no significant difference between the four bed sections. But the intervention targeting the nutritional care had a significantly better effect in bed section IB than in intervention section IIB measured as the extent to which the protein and energy requirements were met. But the quantitative results revealed that the forms designed for assessing the patients nutritional status had been used only to a limited extent. This result was reflected in the results showing that the staff on admission only weighed half of the patients. The outcome of the intervention was probably influenced by the reluctance among the staff in bed section IB to implement the new guidelines, and by the large staff turnover in bed section IIB. Interestingly, the patients' intake of protein- and energy increased significant in bed section IB during the intervention. It cannot be excluded that the focus on the nutritional care coming from an investigator outside of the organization, had led to this paradox that, despite the reluctance identified among the staff, nutritional care was optimised.

**Table 3:** Dietary intake of energy (kJ), estimated requirement for dietary energy (kJ) and degree to which requirement for energy per 24 hours was met before and after intervention. Values are group averages (standard deviation (SD)) unless otherwise stated.

	Medical ward I		Medical ward II	
		
**Before the intervention**	Bed section IA *Status*	Bed section IB *Status*	Bed section IIA *Status*	Bed section IIB *Status*
Dietary intake of energy, kJ/24 hours (SD)	7525 (2927)	6202 (1213)	6938 (2441)	6623 (2352)
Estimated mean of need for dietary energy, kJ/ 24 hours (SD)	8244 (1418)	8177 (1396)	8331 (1533)	7782 (1055)
Estimated need for dietary energy per 24 hours covered in per cent (SD)	**92 **(35)	**77 **(18)	**85 **(30)	**85 **(28)
95 % confidence interval	76–109	60–94	67–103	70–100
	Medical ward I		Medical ward II	
	
**After the intervention**	Bed section IA *Control*	Bed section IB *Intervention*	Bed section IIA *Control*	Bed section IIB *Intervention*
Dietary intake of energy, kJ/24 hours (SD)	5359 (1993)	7267 (2317)	5811 (1851)	5923 (2096)
Estimated mean of need for dietary energy, kJ/ 24 hours (SD)	7396 (1687)	7119 (1619)	7761 (1409)	7810 (1491)
Estimated need for dietary energy per 24 hours covered in per cent (SD)	**74 **(30)	**103 **(24)	**76 **(22)	**76 **(23)
95 % confidence interval	62–87	89–116	62–89	64–89

Patients who were severely mentally or physically impaired were not included in the study of ethical reasons, although they as 'nibblers' did not receive a different form of nutritional care. So the sample is not representative for all the medical patients. If this group of patients had been included the quantitative results probably would have been lower, as described in an other Danish study [28].

The average length of the hospital stay for the patients participating in this study was 23 days. The average length for medical patients in the Aarhus County was six days [16]. This significant difference may be ascribed to the fact that patients hospitalised for less than five days were excluded in this study. On the other hand, mentally or physically impaired patients were not included. The official statistics on the length of hospital stays include a large group of patients who are long-term hospitalised. In this study 27% of the patients were hospitalised for more than four weeks. Long-term hospitalisation demands that particular attention be paid to the problem of weight loss. A 24-hour weight loss reaching 154 gram was found before the intervention, which may, indeed, be regarded as a problem during hospitalisation.

The introduction of new diets made a difference both to the patients and the staff at the bed sections. Thus the 'normal diet' had a lower fat energy percentage than the other diets. This meant that patients had to consume a very sizeable diet in order to cover their energy requirement, which was rarely manageable for patients with reduced appetite. The clinical dietician mentioned that they had most frequently met patients with a poor or reduced appetite and a simultaneous need for a diet with a high nutrient density. This observation is corroborated by observations made by other Danish clinical dieticians [29]. The results of this study indicate that it is hardly appropriate to base nutritional care on recommendations intended for healthy individuals if the staff's nutritional knowledge matches that seen in the present study. The consequences seem to be a deterioration of the nutritional status in an even larger fraction of patients.

The common understanding and recognition of the integration of nutritional care as part of the overall care among all occupational groups is a key prerequisite in an effort to see nutritional care as part of the care for the individual patient [23,30,31]. Another key prerequisite is that responsibility for such care is vested in real professional competence that lies with a single staff group, i.e. that it is backed by knowledge [32]. On this basis it might be possible to establish cooperation and launch a fruitful dialogue.

Nutritional care fell within the competence of the nurses who were therefore able largely to determine to which extent other occupational groups were allowed to contribute with knowledge about nutrition. The clinical dieticians mentioned that they would like a more extensive dialogue with the other care providers about the patients' nutritional status, but these groups did not welcome such cooperation.

One of the nurses in charge found that the intervention had made the nurses pay more attention to nutritional issues including, in particular, patients at increased risk of becoming undernourished. However, it was difficult to translate increased attention into specific nutritional care actions such as recording the patients' nutritional status upon admission by using the special food records. In a study of the relationship between nurses' competences and their knowledge about nutrition and diet in a hospital in the South of England, Lin Perry showed that there was no clear association between the nurses' attitudes, knowledge and actions, as neither knowledge nor attitudes were translated into action [32]. The study also demonstrated discrepancies between what the nurses said they were doing in relation to the patients' nutrition and what was actually documented in the patient records. Perry concluded that nursing care was frustrated by absence or inadequate knowledge among nurses about nutrition or by the failure to communicate such knowledge and a lack of common standards in general.

However, the fact that the staff entertained views on the importance of the food and the food service did not imply that all groups were committed to seeing nutritional care as an element of the overall care effort. And the intervention study was an external project that was not anchored in the bed section's own staff. So that may explain the moderate reluctance to take active part in the study shown by some of the occupational groups [33]. The effect was that the nutritional care was not optimal. Some nurses gave as a reason for this situation that nutritional care was not part of the nursing care and that the time pressures induced by other tasks forced them to give lower priority to nutritional care. In the recommendations of The International Council of Nurses (ICN) the patients' nutritional status is placed second after the first dimension 'ability to breathe' [34]. This implies that a patient's nutritional status is considered an important issue in nursing theory, which is the basis of patient care. This is interesting in the light of the results of the focus group interviews presented here, because it appears that there is no agreement between the guidelines issued by the ICN and the Danish Nurses Organisation as far as the importance of nutrition and the nutritional care the patients receives during hospitalisation is concerned. Several papers in Danish and international nursing journals hence advocate that nurses assume a central role in countering patient under nourishment – a role rarely entertained by nurses today [32,35-37]. Yet, the clinical dieticians and the maids found that the nurses took into account neither their knowledge about patient nutrition in general, nor their knowledge of the individual patient's situation. Paradoxically, however, the nurses still claimed that nutritional care fell mainly within their competence.

The clinical dieticians, the maids and the assistant catering officer reported poor communication between patients, nursing staff and kitchen. But the care staff did not share this view.

The individual bed sections apparently did not have a clear distribution of responsibilities embracing all aspects of nutritional care. On the contrary, the data suggested that dilution of responsibility was accompanied by an element of responsibility evasion.

The degree to which patients' energy and protein requirements are covered undoubtedly varies from hospital to hospital depending on the menus served and the commitment to the nutritional care shown by the care staff and management. However, any food service involves a long chain of tasks and work processes reaching from the kitchen to the patient, and the food service is essentially organised in the same way and priorities are generally the same in all Danish hospitals. It is therefore likely that the problems associated with insufficient nutritional care are of a similar nature in Danish hospitals and some European hospitals [38]. The perspective for further investigation could be a Health Technology Assessment (HTA) to evaluate the aspect of the patients, the organisation and the economy of the nutritional care of medical inpatients.

## Conclusion

The average intake of energy and protein among hospitalised medical patients did not cover their requirements. Prior to intervention, food ingested during hospitalisation on average met 72% of the patients' protein and 85% of their energy requirement. After changing the diets from 'unrestricted diet' to 'normal diet' and 'hospital diet', the diet on average met 61% of the control patients' protein and 75% of their energy requirements. Intervention allowed a significantly better satisfaction of the patients' protein and energy requirements at one of the intervention sections using standard hospital food. However, the implementation of procedures focusing on nutritional care appeared to be difficult, especially at bed sections with a large staff turnover. Consequently, the results of the study call attention to the existence of barriers to efforts aimed at improving the nutritional care of patients.

Introduction of nutritional care as part of the overall care met with barriers among the care providers. Focus group interviews identified these barriers as lack of time, lack of knowledge, lack of contact with resource agents concerning nutrition, lack of commitment, resistance towards a additional perceived workload and resistance towards providing service to the patients. Care providers who wished to provide individual nutritional care saw the very organisation of the food service as an obstacle to their freedom of action and flexibility. The effect of this was that it was difficult to accommodate individual patients' requirements.

Occupational groups involved in nutritional care worked on the basis of different perceptions, had no shared target and no clear division of responsibility. Improvement of the nutritional care requires that focus be directed towards the final link in the food service chain.

This study showed that nutritional care was a subordinate rather than a coordinate element in the overall care effort. The failure of coordination hinged on dimensions of organisation, knowledge and resource utilisation and it significantly affected the degree to which patients' nutritional requirements were met. An increase in the priority given to nutritional care by central hospital management and a concomitant general change in attitude towards nutritional care is needed and is probably a precondition for achieving a level of sufficient nutrition among hospitalised patients.

## Authors' contributions

Karin O. Lassen carried out the research design, the fundraising, coordination of organisational communication, food record planning and implementation, performed the data analysis, drafted the manuscript and is the guarantor of the manuscript. Filip Kruse participated in the designing the focus group interviews, in the data analysis, and as author of the manuscript. Merete Bjerrum participated in the data analysis and as author of the manuscript. Lillian Jensen participate in the planning of the food records, performed the calculation of protein and energy intake and participate in the discussion of the manuscript. Kjeld Hermansen contribute to organisational support and discussion of research design and manuscript. All authors read and approved the final manuscript.

## Competing interests

None declared.
